# Virginia Medical College

**Published:** 1858-07

**Authors:** 


### Virginia Medical College.

We have received the catalogue of the Medical College of Virginia, at Richmond, Va., for the Session '57–'58, and the announcement of '58–'59, with a catalogue of all the graduates of the school.

From this catalogue, we learn that they had in attendance at the last course 60 Matriculates, and conferred the degree upon 21 gentlemen, and report 416 as the whole number of graduates. We perceive that this Institution has lengthened its term to five months, commencing the 4th of October, and closing the 1st of March.

It has always seemed to us that Richmond was one of the very best points for a successful Medical School.

From the facilities offered by the Medical College of Virginia, we would desire to commend it to the attention of those who wish to obtain a complete medical education in a Southern city.

Richmond not only furnishes every necessary facility for Medical Instruction, but as a place of residence, is unquestionably the most desirable city in the United States, and the student will find there a most agreeable place of sojourn.

